# Construction of a Fluorescence‐Based Logic Gate Seeing the Effect of Perchlorate Ions on Hemicyanine Dye–*β*‐Cyclodextrin Complexes to Certify Safe Drinking Water

**DOI:** 10.1002/open.202500152

**Published:** 2025-05-04

**Authors:** Anusha C. M., Shalini Dyagala, Sairathna Choppella, Mahesh Kumar Ravva, Subit Kumar Saha

**Affiliations:** ^1^ Department of Chemistry Birla Institute of Technology & Science (BITS) Pilani Hyderabad Campus Hyderabad Telangana 500078 India; ^2^ Department of Chemistry SRM University‐AP Amaravati 522240 India

**Keywords:** *β* ‐CD, analytical methods, DASPC22, fluorescence spectroscopy, perchlorate ions (ClO_4_^−^)

## Abstract

Perchlorate ions (ClO_4_^−^) are prevalent contaminants in the surface, and drinking water that disrupt thyroid function by competitively inhibiting the sodium‐iodide symporter (NIS), posing significant health risks. Here, fluorescence‐based logic gates have been constructed by leveraging the binding interactions between a hemicyanine dye, 4‐[4‐(dimethylamino)‐styryl]‐1‐docosylpyridinium bromide (DASPC22) and *β*‐cyclodextrin (*β*‐CD) that could be useful to know whether ClO_4_^−^ ions in water are within the toxicity range or not. In aqueous media, DASPC22 forms nonfluorescent H‐aggregates, but fluorescence is enhanced upon forming host‐guest inclusion complexes with *β*‐CD. At low ClO_4_^−^ ions concentrations, fluorescence intensity further increases due to enhanced complex stability through hydrogen bonding. ONIOM‐based quantum chemical calculations have supported this phenomenon. The enhancement of fluorescence intensity of DASPC22 in the presence of *β*‐CD and a low concentration of ClO_4_^−^ ions leads to the construction of a YES logic gate that would enable one to quantify ClO_4_^−^ ions’ toxicity range in water. Dual‐input‐single‐output AND and INHIBIT logic gates with low and high concentrations of ClO_4_^−^ ions, respectively, have also been constructed. The present system could be useful in addressing safety concerns related to perchlorate contamination of water.

## Graphical abstract

A method to detect perchlorate ions and certify safe drinking water by constructing a fluorescence‐based logic gate considering the binding of a hemicyanine dye with *β*‐CD is presented.

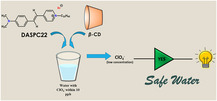

## 1 Introduction

In many countries, the surface and drinking water is found to contain perchlorate.^[^ ^1^ ^]^ In addition to the release of perchlorate from military and defence operations, there are some natural sources like potash ores and fertilizers. The major toxic effect of perchlorate ions (ClO_4_^−^) on humans is the disruption of thyroid function. It competitively inhibits the sodium‐iodide symporter (NIS), which is a membrane glycoprotein responsible for the uptake of iodide into the thyroid and other organs.^[^ ^2^, ^3^ ^]^ Perchlorate is a potent inhibitor of this transporter, with an affinity ≈30 times higher than that of iodide. Perchlorate has been a chemical substance of concern due to its presence in drinking water aquifers and known toxicity. A maximum concentration of perchlorate was found to be 44.2 ppb in a study conducted after a display of fireworks.^[^ ^4^ ^]^ Though a maximum level of contamination is not set, but a limit of 6 ppb is suggested by Health Canada.^[^ ^1^ ^]^ Guidelines ranging from 1 to 18 ppb for ClO_4_^−^ ions have been implemented by different states of the United States.^[^ ^1^ ^]^ The chromatographic method is reported to be primarily used to measure the perchlorate.^[^ ^1^ ^]^ A number of methods are reported for the removal of ClO_4_^−^ ions from the water, such as filtration by a membrane, and anion exchanger, including various chemical and biological processes.^[^ ^1^ ^]^

**Scheme 1:**
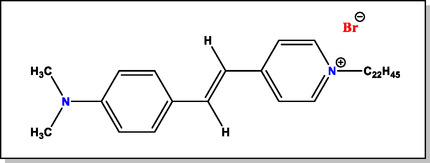
The molecular structure of DASPC22.

In the present work, we have described the construction of logic gates based on the effect of ClO_4_^−^ ions on the binding strengths of a hemicyanine dye, 4‐[4‐(dimethylamino)‐styryl]‐1‐docosylpyridinium bromide (DASPC22) (**Scheme** **1**) with *β*‐cyclodextrin (*β*‐CD) with its possible application toward finding whether ClO_4_^−^ ions in water are in toxic range or not. DASPC22 is a hemicyanine dye molecule having *N,N*‐dimethyl amino group as a donor and docosylpyridinium group as an acceptor.^[^ ^5^ ^]^ In a polar medium, fluorescence occurs from a non‐twisted intramolecular charge transfer (Non‐TICT) state. The TICT state formation reduces the fluorescence quantum yield of DASPC22. DASPC22 molecules are found to be nonfluorescent in an aqueous medium, forming H‐aggregates.^[^ ^5^ ^]^ However, the aggregation of dye molecules can be controlled by inclusion with the *β*‐CD molecules.^[^ ^5^, ^6^ ^]^ Once inclusion complexes are formed, a major portion of the dye molecules exist in the monomeric forms, and therefore, the fluorescence intensity is enhanced. In our earlier study,^[^ ^5^ ^]^ we have demonstrated that simple inclusion/host‐guest complexes of 1:2 (DASPC22:*β*‐CD) stoichiometry between DASPC22 and *β*‐CD are formed up to 2.7 mM of *β*‐CD. However, beyond this concentration, *β*‐CD molecules are threaded along the hydrocarbon tail of DASPC22, giving nanotubes and secondary aggregates of nanotubes.^[^ ^5^, ^7^, ^8^, ^9^, ^10^, ^11^, ^12^ ^]^ As a result, a great extent of dye molecules exist in the monomeric form, showing a large increase in fluorescence intensity. A ≈350‐fold increase in fluorescence intensity of DASPC22 (5.0 μM) has been noted in the presence of 8.0 mM of *β*‐CD in a phosphate buffer solution of pH 7.4.^[^ ^5^ ^]^ This high extent of enhancement in fluorescence intensity of DASPC22 upon binding with the *β*‐CD molecules makes it a very sensitive dye that tempted us to explore it further.

The hydrophobic interactions are mainly responsible for the formation of complexes between hydrophobic host and guest.^[^ ^7^, ^8^, ^9^, ^10^, ^11^, ^12^, ^13^ ^]^ It has been reported that the salting‐in and salting‐out agents have roles in the stability of host–guest complexes.^[^ ^13^ ^]^ The solubility of hydrocarbon substances in water can be altered by the addition of cations or anions depending on their sizes and polarizabilities. Cl^−^ and Li^+^, being small ions, act as salting‐out agents and reduce the solubility of hydrocarbons. Therefore, the binding strength between the hydrophobic host and guest molecules increases, and the stability of the host–guest complexes is enhanced. On the contrary, I^−^, ClO_4_^−^, and Gn^+^, that is, guanidinium ions being large ions, act as salting‐in agents, enhancing hydrocarbons’ water solubility and resulting in decreased stability of the host‐guest complexes.^[^ ^14^ ^]^

In our earlier study,^[^ ^5^ ^]^ we have noticed the dual behavior of a Hofmeister series of ions, F^−^, Cl^−^, I^−^, and ClO_4_^−^, in the formation of stable complexes between hydrophobic host and guest molecules. In the low concentration range, these ions act like salting‐out agents, while in a comparatively higher concentration range, they behave like salting‐in agents. Though dual behavior has been noted for all these ions, but the most notable dual behavior has been observed in the case of ClO_4_^−^. It has been found that the fluorescence intensity of 5.0 μM of DASPC22 in 8.0 mM of *β*‐CD further increases upon the addition of ClO_4_^−^ ions up to 8.1 ppb, and then the fluorescence intensity decreases with the addition of more amount of ClO_4_^−^ ions. It has been explained that in the low concentration range, a ClO_4_^−^ ion interacts with two molecules of neighboring *β*‐CD, taking part in the nanotube formation through hydrogen bonding.^[^ ^5^, ^7^ ^]^ It has also been reported that the host–guest complexes become stronger at a low concentration range of ClO_4_^−^ and I^−^ ions due to these ions’ closer contact with the positively charged dye compared to the other two ions, F^−^ and Cl^−^. The greater extent of binding interactions between *β*‐CD and DASPC22 at a low concentration of ClO_4_^−^ ions compared to I^−^ ions is due to ClO_4_^−^ ions’ extra effect, that is, the anchoring with –OH groups of *β*‐CD molecules.^[^ ^15^ ^]^ It is suggested that hydrogen bonding occurs between the secondary —OH groups bonded with the *β*‐CD's larger rim and the oxygen atoms of a ClO_4_^−^ ion.^[^ ^5^ ^]^ As a result, the binding strength between *β*‐CD and DASPC22 molecules is enhanced, so the fluorescence intensity of the dye increases. Beyond, 8.1 ppb, the ClO_4_^−^ ions act as salting‐in agents, and therefore, the dye's fluorescence intensity is quenched due to the dissociation of host–guest complexes and also the competition between DASPC22 and ClO_4_^−^ ions to bind with the cavities of *β*‐CD molecules.^[^ ^5^, ^13^, ^15^, ^16^ ^]^

Various chemosensors have been developed to detect toxic ions such as Cu^2^^+^,^[^ ^17^ ^]^ Sn^4^^+^ ,^[^ ^18^ ^]^ and Cr_2_O_7_^2^^−^,^[^ ^18^ ^]^ using dual‐channel fluorescence methods and functionalized nanoparticles. Recent studies have demonstrated the use of *β*‐CD‐functionalized silver nanoparticles^[^ ^19^ ^]^ and naphthoquinone‐based sensors for selective ion detection,^[^ ^20^ ^]^ highlighting their potential for environmental monitoring and biomedical applications, including cancer cell imaging. These advances have motivated the current work, which utilizes DASPC22, in conjunction with *β*‐CD for the detection of ClO_4_^−^ ions in water.

In the present work, we have first shown the role of ClO_4_^−^ ions in the stability of host–guest complexes between *β*‐CD and DASPC22 by quantum chemical calculations using ONIOM methods and recording UV‐Vis absorption spectra of ClO_4_^−^ ions. The phenomena of an increase in the stability of host–guest complexes resulting in an increase in fluorescence intensity in the low concentration range of ClO_4_^−^ ions, and a decrease in the stability of complexes with a quenching of fluorescence intensity at a high concentration range of ClO_4_^−^ ions have been utilized to construct different logic gates. One of these logic gates can be applied to know whether ClO_4_^−^ ions present in the water are outside the toxic range, so the water is drinkable. To the best of the authors’ knowledge, there is no report on detecting ClO_4_^−^ ions in the toxic range with the help of a change in fluorescence intensity of a dye, here, DASPC22 complexed with *β*‐CD.

## 2 Results and Discussion

### 2.1 Binding of DASPC22 with *β*‐CD: Quantum Chemical Calculations

In a polar medium, the fluorescence of DASPC22 is quenched due to the formation of H‐aggregates.^[^ ^5^ ^]^ In an earlier study, it was observed that the fluorescence intensity of DASPC22 is increased upon binding with the *β*‐CD due to the existence of a monomeric form of the dye. Below the critical aggregation concentration (2.7 mM) of *β*‐CD, simple inclusion complexes between DASPC22 and *β*‐CD of 1:2 stoichiometry are formed, and fluorescence intensity is increased compared to a polar medium. The binding constant for this inclusion complex with 1:2 stoichiometry was found to be 2.53 × 10^6^ M^−2^. Above 2.7 mM, *β*‐CD molecules are gradually threaded along the tail of DASPC22, as a result, the extended nanotubes are formed, and the dye molecules remain as monomers. Therefore, fluorescence intensity is significantly increased. The fluorescence intensity is further increased in the presence of a low concentration of ClO_4_^−^ ions. Earlier, it has been proposed that in the low concentration range, a ClO_4_^−^ ion interacts with two molecules of neighboring *β*‐CD taking part in the nanotube formation through hydrogen bonding,^[^ ^5^, ^7^ ^]^ and therefore, binding strength between the host and the guest is enhanced. Not only fluorescence spectroscopic measurements but to describe the strength of interactions between DASPC22 and *β*‐CD and the effect of ClO_4_^−^ ions, here, for the first time, the quantum chemical calculations have been performed to present the geometry, interaction energy, and the effect of low concentration of ClO_4_^−^ ions on it, applying a two‐level ONIOM method that has been described below.

**Figure 1:**
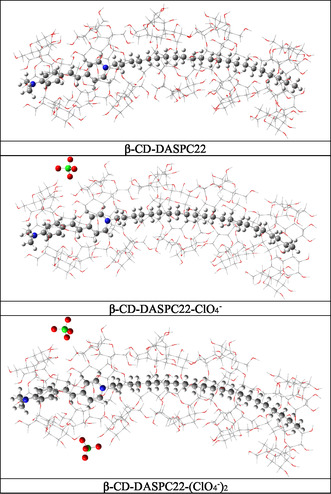
Optimized geometries of *β*‐CD‐DASPC22, *β*‐CD‐DASPC22‐ClO_4_^−^, and *β*‐CD‐DASPC22‐(ClO_4_^−^)_2_ complexes obtained at the ωB97XD/6‐31g(d,p):UFF level of theory.

In order to understand the impact of ClO_4_^−^ ions on the stability of *β*‐CD‐DASPC22 complexes, geometry optimizations have been carried out both in the presence and absence of ClO_4_^−^ ions. The strength of interactions between the *β*‐CD nanotube and DASPC22 molecule has been estimated using the optimized geometries. **Figure** **1** depicts the optimized geometries of *β*‐CD‐DASPC22 complexes in the absence of ClO_4_^−^ ions (*β*‐CD‐DASPC22), in the presence of one ClO_4_^−^ ion (*β*‐CD‐DASPC22‐ClO_4_^−^), and two perchlorates (*β*‐CD‐DASPC22‐(ClO_4_^−^)_2_) determined at the QM/MM level of theory. The calculated interaction energies between *β*‐CD and the DASPC22 molecules are presented in **Table** **1**. While calculating interaction energies between cationic DASPC22 molecule and *β*‐CD rings, two rings of *β*‐CD units that interact with *N,N*‐dimethyl amino groups of the cationic DASPC22 molecule have been considered to minimize computational cost. The calculated interaction energies indicate that increasing ClO_4_^−^ ions leads to stronger interactions between the DASPC22 molecule and *β*‐CD rings. Table 1 shows that adding one additional perchlorate ion enhances the interaction between *β*‐CD and the DASPC22 molecule by ≈2.00 kcal mol^−1^.

**Table 1:** Counterpoise corrected interaction energies of the complexes calculated at ωB97XD/6‐31g(d,p) level of theory.

Complex	Interaction energy [kcal mol^−1^]
β‐CD‐DASPC22	–68.14
β‐CD‐DASPC22‐ClO_4_^−^	–69.93
β‐CD‐DASPC22‐(ClO_4_^−^)_2_	–71.30

**Figure 2:**
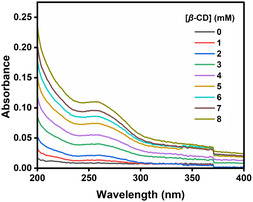
Absorption spectra of ClO_4_^−^ ion (500 ppb) as a function of the concentration of *β*‐CD.

**Figure 3:**
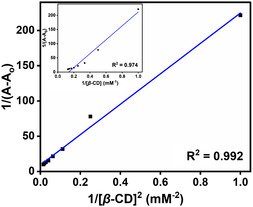
Benesi−Hildebrand plot for ClO_4_^−^ ion (500 ppb) and *β*‐CD complex for a 1:2 stoichiometry. Inset: Benesi−Hildebrand plot for a 1:1 stoichiometry.

The binding of the ClO_4_^−^ ions with the *β*‐CD has also been studied by recording the absorption spectrum of the ClO_4_^−^ ions at different concentrations of the *β*‐CD. The ClO_4_^−^ ions’ absorption peak appears at 260 nm. **Figure** **2** shows how the absorption spectrum of ClO_4_^−^ ions is changing with increasing concentration of *β*‐CD from 0 to 8 mM. The binding constant for both 1:1 and 1:2 stoichiometry has been calculated using the Benesi–Hildebrand equations, Equation (1) and (2), respectively from this data:

(1) $$\frac{1}{A-A_{o}}=\;\frac{1}{A_{m}-A_{o}}+\frac{1}{\text{K }\left[\text{CD}\right](A_{m}-A_{o})}$$

(2) $$\frac{1}{A-A_{o}}=\;\frac{1}{A_{m}-A_{o}}+\frac{1}{K^{\prime }{\left[\text{CD}\right]}^{2}(A_{m}-A_{o})}$$

where A_o_ and A represent the absorbance in the absence and presence of *β*‐CD, respectively. A_m_ = limiting absorbance. [*β*‐CD] = experimental concentration of *β*‐CD. The association constants following 1:1 and 1:2 stoichiometries are denoted by *K* and *K* ^′^, respectively. The Benesi−Hildebrand plot for the complex of the ClO_4_^−^ ions, and *β‐*CD with 1:2 stoichiometry is shown in **Figure** **3** (correlation coefficient = 0.992). It does not give a good correlation for 1:1 stoichiometry, but it gives a better correlation with the 1:2 stoichiometry, and the binding constant for 1:2 stoichiometry is calculated as 4.25 × 10^4^ M^−2^. These results nicely support the stability of the DASPC22‐*β*‐CD complex through the interaction of one ClO_4_^−^ ion with two *β*‐CD molecules.

### 2.2 Effect of Perchlorate Ions on the Binding of DASPC22 with *β*‐CD

**Figure 4:**
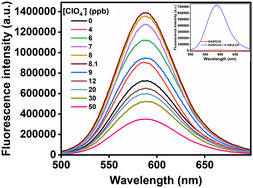
Fluorescence spectra of DASPC22 in the presence of 8.0 mM of *β*‐CD and different concentrations of ClO_4_^−^ ions in the aqueous media at pH 7.4. [DASPC22] = 5.0 μM. *λ*_ex_ = 423 nm.

**Figure 5:**
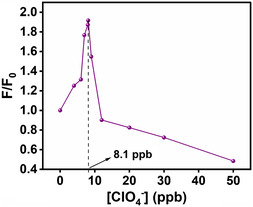
Fluorescence intensity ratios of DASPC22 in 8.0 mM of *β*‐CD with different concentrations of ClO_4_^−^ ions in the aqueous media at pH 7.4. [DASPC22] = 5.0 μM, *λ*_ex_ = 423 nm, *λ*_em_ = 588 nm.

**Figure** **4** shows fluorescence spectra of DASPC22 in the presence of 8.0 mM of *β*‐CD and different concentrations of ClO_4_^−^ ions in the aqueous media at pH 7.4. This experiment is different from our earlier reported data^[^ ^5^ ^]^ in terms of the fact that here, instead of phosphate buffer, simple acid and base (H_2_SO_4_/NaOH) have been used to adjust the pH of 7.4, as we have to deal with drinking water. The inset of this figure presents fluorescence spectra of DASPC22 in water and in a solution containing only 8.0 mM of *β*‐CD. There is a ≈350‐fold increase in fluorescence intensity of DASPC22 in the presence of 8.0 mM of *β*‐CD compared to the dye in water. As mentioned earlier, in the presence of 8.0 mM of *β*‐CD, the cyclodextrin molecules encapsulate the chromophore unit and are threaded along the hydrocarbon tail of the dye. As a result, it is difficult for the dye molecules to form H‐aggregates. They remain in the monomeric state. That is why the fluorescence intensity is largely increased. **Figure** **5** displays fluorescence intensity ratios, *F*/*F*_o_ (where *F* and *F*_o_ are fluorescence intensities of DASPC22 in the presence and absence of ClO_4_^−^ ions, respectively) of DASPC22 at *λ*_em_ = 588 nm in the presence of 8.0 mM of *β*‐CD and different concentrations of ClO_4_^−^ ions obtained from the fluorescence spectra given in Figure 4. It can be seen from Figure 5 that in the presence of 8.1 ppb concentration of ClO_4_^−^ ions, the fluorescence intensity is further increased by 1.9‐fold compared to 8.0 mM of *β*‐CD. As mentioned previously, a ClO_4_^−^ ion provides an anchoring site by which hydrogen bonds are formed with two molecules of neighboring *β*‐CD. As a result, the *β*‐CD‐DASPC22 complex becomes stronger compared to a system without ClO_4_^−^ ions, showing an effect similar to that done by salting‐out agents. That is why the fluorescence intensity is enhanced in the presence of ClO_4_^−^ ions. However, at a concentration higher than 8.1 ppb, ClO_4_^−^ ions act as salting‐in agents, and therefore, the *β*‐CD‐DASPC22 complexes dissociate, resulting in a decrease in fluorescence intensity of the dye as the dye molecules come out of the nanotubular cavities of *β*‐CD molecules. Quantum chemical calculations and spectroscopic measurements discussed earlier support the increase in the strength of the complex between *β*‐CD and DASPC22 in the presence of a few ClO_4_^−^ ions.

### 2.3 Logic Gates Construction

Based on the fluorescence properties of the dye, DASPC22 in an aqueous medium with one or two chemical inputs and fluorescence intensity of the dye at a wavelength as a single optical output, several logic gates have been constructed. A threshold value has been set up to the fluorescence intensity to convert analog intensity data into binary digits, applying one/two chemical inputs. The binary digits 0 and 1 were assigned to the fluorescence intensity below (fluorescence OFF) and above (fluorescence ON) the threshold value, respectively. Constructions of different logic gates are given below.

#### 2.3.1 Single‐Input‐Single‐Output: Logic Gate 1

**Figure 6:**
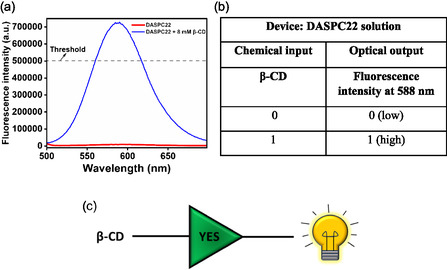
a) Fluorescence spectra of 5.0 μM of DASPC22 with and without 8.0 mM *β*‐CD in an aqueous solution at pH 7.4, b) truth table, and c) YES logic gate representation.

Here, an aqueous solution of DASPC22 at 5.0 μM concentration at pH 7.4 is treated as a device. Chemical input is an aqueous solution of 0.0 or 8.0 mM of *β*‐CD. Optical output is the fluorescence intensity of DASPC22 at *λ*_em_ = 588 nm. **Figure** **6**a displays fluorescence spectra of 5.0 μM of an aqueous solution of DASPC22 at pH 7.4 in the absence and presence of 8.0 mM of *β*‐CD. The threshold value is the fluorescence intensity of 500,000. Based on the fluorescence data, the truth table created is shown in Figure 6b. As per the truth table, the logic gate constructed is YES, which is shown in Figure 6c.

#### 2.3.2 Single‐Input‐Single‐Output: Logic Gate 2

**Figure 7:**
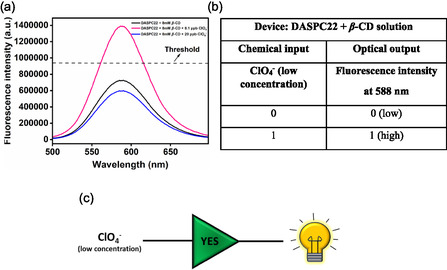
a) Fluorescence spectra of 5.0 μM of DASPC22 in the presence of 8.0 mM *β*‐CD and 8.1 ppb (low concentration) and 20.0 ppb (high concentration) of ClO_4_^−^ ions in aqueous solution at pH 7.4, b) truth table and c) YES logic gate representation.

For this logic gate, the aqueous solution of 5.0 μM of DASPC22 in the presence of 8.0 mM of *β*‐CD is considered as the device, and the low concentration of ClO_4_^−^ ions (up to 8.1 ppb) is the single chemical input. As discussed earlier, the fluorescence intensity of DASPC22 increases up to 8.1 ppb concentration of ClO_4_^−^ ions in the presence of 8.0 mM of *β*‐CD due to the ternary complex formation between one ClO_4_^−^ ion and two molecules of *β*‐CD through hydrogen bonding showing a kind of salting‐out effect. Above 8.1 ppb concentration of ClO_4_^−^ ions, the salting‐in effect is in place, and therefore, the fluorescence intensity of the dye starts decreasing. **Figure** **7**a presents fluorescence spectra of 5.0 μM of DASPC22 binding with 8.0 mM of *β*‐CD in the absence and presence of 8.1 ppb (low concentration) and 20.0 ppb (high concentration) of ClO_4_^−^ ions. Figure 7b displays a truth table based on the fluorescence intensity at *λ*_em_ = 588 nm above and below the threshold intensity set as 940,000. According to this truth table, the logic gate constructed is YES, as shown in Figure 7c. This logic gate can be used as described below.

**Figure 8:**
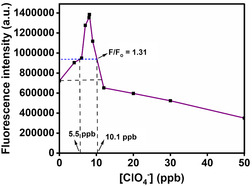
Fluorescence intensity of DASPC22 in 8.0 mM of *β*‐CD with different concentrations of ClO_4_^−^ ions in the aqueous media at pH 7.4. [DASPC22] = 5.0 μM *λ*_ex_ = 423 nm. *λ*_em_ = 588 nm.

**Figure 9:**
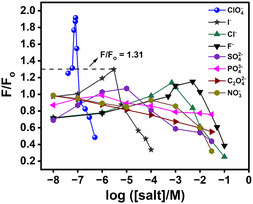
*F*/*F*_o_ of 5.0 μM DASPC22 in 8.0 mM *β*‐CD in the presence of a range of each ion concentration. *λ*_ex_ = 423 nm. *λ*_em_ = 588 nm.

The ClO_4_^−^ ions’ major toxic effect in humans is the disruption of thyroid function. It competitively inhibits the sodium‐iodide symporter (NIS), which is a membrane glycoprotein responsible for the uptake of iodide into the thyroid and other organs.^[^ ^2^, ^3^ ^]^ Perchlorate is a potent inhibitor of this transporter, with an affinity ≈30 times higher than that of iodide. Different agencies^[^ ^1^ ^]^ recommend the desirable ClO_4_^−^ ions concentration in drinking water should be within 1–18 μg L^−1^ (1–18 ppb). Figure 5, given above, shows that the fluorescence intensity of DASPC22 increases up to 8.1 ppb ClO_4_^−^ ions in the presence of 8.0 mM of *β*‐CD and then decreases. **Figure** **8** displays the fluorescence intensity of 5.0 μM of DASPC22 in 8.0 mM of *β*‐CD with varying concentrations of ClO_4_^−^ ions. At 11.5 ppb of ClO_4_^−^ the intensity becomes the same as that in the presence of only 8.0 mM of *β*‐CD. It has been described below (**Figure** **9**) that for iodide ions (I^−^), the maximum intensity ratio can go up to 1.31. Whereas for ClO_4_^−^, the maximum intensity ratio goes up to 1.91. Therefore, if a threshold fluorescence intensity value of the dye is set as 940,000, that is, equivalent to *F/F*_o_ = 1.31 in Figure 5, then at all concentrations of ClO_4_^−^ ions within 5.5 to at least 10.1 ppb, the logic gate constructed will be nothing but YES. The desirable ClO_4_^−^ ions concentration in drinking water should not exceed 18 ppb. To be on the safer side, the maximum value can be set as 10.0 ppb. An acceptable concentration of ClO_4_^−^ ions lies on both sides of the peak. It can be stated that any concentration of ClO_4_^−^ ions for which the fluorescence intensity ratio, *F/F*_o_, is greater than 1.31, is safe for the water to drink. The concentration of ClO_4_^−^ ions with *F/F*_o _= 1.31 value falls within 5.5 to 10.1 ppb which is within the non‐toxicity range of ClO_4_^−^ ions. Therefore, as per the present ClO_4_^−^ ions detection system, the lower limit of detection (LLOD) is set as 5.6 and the higher limit of detection (HLOD) is set as 10.0 ppb. Thus, the present logic gate can be used to certify whether drinking water is safe from ClO_4_^−^ ions or not. Some more experiments have been done to see the effect of the presence of other ions along with ClO_4_^−^ ions.

**Figure 10:**
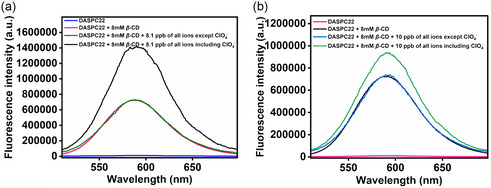
a) Fluorescence spectra of 5.0 μM of DASPC22 in 8.0 mM of *β*‐CD in the presence of 8.1 ppb of all ions together with and without ClO_4_^−^ ions, and b) fluorescence spectra of 5.0 μM of DASPC22 in 8.0 mM of *β*‐CD in the presence of 10.0 ppb of all ions together with and without ClO_4_^−^ ions. *λ*_ex_ = 423 nm.

**Figure 11:**
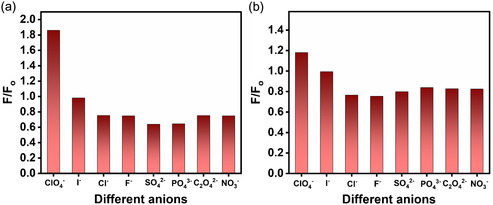
Histogram for fluorescence intensity ratios (*F*/*F*_o_) of 5.0 μM of DASPC22 in 8.0 mM of *β*‐CD in the presence of a) 8.1 ppb and b) 10.0 ppb of different anions in aqueous solutions at pH 7.4. *λ*_ex_ = 423 nm. *λ*_em_ = 588 nm.

The ions, ClO_4_^−^, I^−^, Cl^−^, F^−^, SO_4_^2−^, PO_4_^3−^, C_2_O_4_^2−^, and NO_3_^−^, fall under the Hofmeister series^[^ ^5^, ^21^, ^22^, ^23^ ^]^ To check the possibility of the presence of these ions, along with ClO_4_^−^ ions, and the effect on the fluorescence intensity of DASPC22 in the presence of *β*‐CD, some control experiments have been done. Figure 9 displays the fluorescence intensity ratio, *F/F*_o_ (where *F* and *F*_o_ are fluorescence intensities of DASPC22 in the presence and absence of ions, respectively) of 5.0 μM DASPC22 in 8.0 mM *β*‐CD in the presence of a range of each ion concentration. Similar trends were noticed earlier^[^ ^5^ ^]^ for ClO_4_^−^, I^−^, Cl^−^, and F^−^ ions in phosphate buffer medium with a change in the range of concentrations to show salting‐in and salting‐out effects. As can be seen here, the salting‐out effect of ions other than ClO_4_^−^ ions mostly starts after the salting‐in effect of ClO_4_^−^ ions is in place. **Figure** **10**a displays fluorescence spectra of 5.0 μM of DASPC22 in 8.0 mM of *β*‐CD in the presence of 8.1 ppb of all ions with and without ClO_4_^−^ ions. Figure 10b presents fluorescence spectra of 5.0 μM of DASPC22 in 8.0 mM of *β*‐CD in the presence of 10.0 ppb of all ions together with and without ClO_4_^−^ ions. It can be seen that in both cases, the fluorescence intensity of DASPC22 in 8.0 mM of *β*‐CD is almost the same as that in the presence of all ions except ClO_4_^−^ ions. However, in the presence of each of 8.1 and 10.0 ppb of ClO_4_^−^ ions in the solution containing all other ions, the fluorescence intensity is higher than that in the presence of only 8.0 mM of *β*‐CD. The intensity difference is less in the presence of 10.0 ppb of ClO_4_^−^ ions due to the salting‐in effect as compared to the salting‐out effect in the presence of 8.1 ppb of the ions. From these results, it is confirmed that the fluorescence intensity of the dye is increased only in the presence of ClO_4_^−^ ions with respect to that in 8.0 mM of *β*‐CD without any ions. **Figure** **11**a,b display the histogram for fluorescence intensities of 5.0 μM of DASPC22 at *λ*_em_ = 588 nm in the presence of 8.1 and 10.0 ppb of each of different anions and 8.0 mM of *β*‐CD. It can be seen from these figures that for ClO_4_^−^ ions, the fluorescence intensity is the highest.

To check the unknown water sample, DASPC22 of concentration of 5.0 μM followed by *β*‐CD of concentration 8.0 mM to be added to the sample. The fluorescence spectrum of this sample is to be recorded. From this spectrum, the *F* at *λ*_em_ = 588 nm is to be measured. The fluorescence spectrum of a pure water sample containing 5.0 μM DASPC22 and 8.0 mM of *β*‐CD will be recorded, and *F*_o_ at *λ*_em_ = 588 nm will be calculated. These two values will give *F*/*F*_o_ at *λ*_em_ = 588 nm for the unknown sample. Figure 9 displays that next to ClO_4_^−^ ions, the intensity ratio that can be increased is due to I^−^ ions. The maximum intensity ratio goes up to 1.31 in the presence of I^−^ ions. Except for ClO_4_^−^ and I^−^ ions, for the rest of the anions, the maximum intensity ratio is shown to be less than 1.31. Therefore, if the *F*/*F*_o_ at *λ*_em_ = 588 nm of the unknown sample is higher than 1.31, then it must be due to the presence of ClO_4_^−^ ions. With this intensity ratio, the concentration of ClO_4_^−^ ions in the water sample lies in the range of 5.5–10.1 ppb and is suitable for drinking. So, the lower limit of detection (LLOD) for the present system is set as 5.6 ppb, and the higher limit of detection (HLOD) is set as 10.0. The development of the present detection system is based on the effect of ClO_4_^−^ ions on the change in fluorescence intensity of DASPC22 complexed with *β*‐CD due to enhanced binding strength in the presence of ClO_4_^−^ ions through hydrogen bonding, and a similar mechanism is not applicable for cations, therefore, study with toxic cations is redundant here.

The obtained results have been compared with the existing literature reports employing various methods for ClO_4_^−^ ions detection (Table S1, Supporting Information). The novelty of the present work lies in the development of a simple, cost‐effective, and highly selective fluorescence‐based platform utilizing the DASPC22–*β*‐CD complex system. This approach is validated through number of experiments related to the interactions of ClO_4_^−^ ions with the complex in the absence and presence of various other ions. In contrast to several reported techniques that depend on expensive, time‐intensive, and instrument‐heavy methodologies such as LC/ESI‐MS/MS,^[^ ^24^ ^]^ chromatography,^[^ ^25^, ^26^ ^]^ electrochemiluminescence^[^ ^27^ ^]^ or biosensors,^[^ ^28^ ^]^ our system provides a rapid optical response with a detection limit in the low ppb range, rendering it highly suitable for practical environmental monitoring of ClO_4_^−^ ions in drinking water.

#### 2.3.3 Single‐Input‐Single‐Output: Logic Gate 3

**Figure 12:**
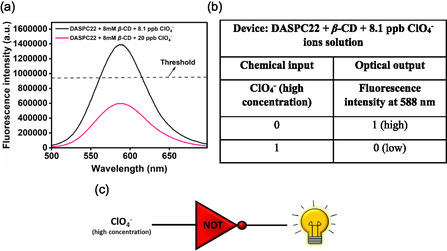
a) Fluorescence spectra of DASPC22 in 8.0 mM *β*‐CD in the presence of 8.1 and 20.0 ppb of ClO_4_^−^ ions, b) truth table, and c) NOT logic gate representation.

In this logic gate, the device is the aqueous solution of 5.0 μM of DASPC22 in the presence of 8.0 mM of *β*‐CD and low concentration of ClO_4_^−^ ions (8.1 ppb). The high concentration of ClO_4_^−^ ions (20.0 ppb) is the chemical input. Single optical output is the fluorescence intensity of DASPC22 at *λ*_em_ = 588 nm. At a concentration higher than 8.1 ppb, the ClO_4_^−^ ions start to show a salting‐in effect in the presence of 8.0 mM of *β*‐CD. **Figure** **12**a presents the fluorescence spectra of 5.0 μM of DASPC22 in 8.0 mM of *β*‐CD in the presence of 8.1 and 20.0 ppb of ClO_4_^−^ ions. Figure 12b represents the truth table obtained after setting a threshold value for the fluorescence intensity of 940,000. Based on the chemical input and optical data, the NOT logic gate is constructed as shown in Figure 12c.

#### 2.3.4 Dual‐Input‐Single‐Output: Logic Gate 4

**Figure 13:**
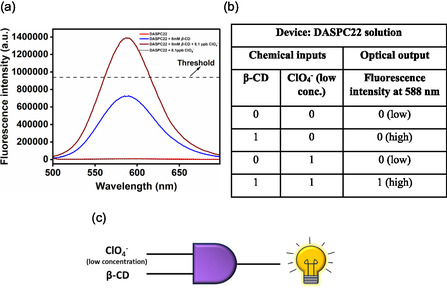
a) Fluorescence spectra of DASPC22, DASPC22 + 8.1 ppb ClO_4_^−^ ions, DASPC22 + 8.0 mM *β*‐CD, DASPC22 + 8.0 mM *β*‐CD + 8.1 ppb ClO_4_^−^ ions, b) truth table, and c) AND logic gate representation.

Here aqueous solution of DASPC22 at 5.0 μM concentration at pH 7.4 is treated as a device. Chemical inputs are 8.0 mM of *β*‐CD and 8.1 ppb of ClO_4_^−^ ions. Single optical output is the fluorescence intensity of DASPC22 at *λ*_em_ = 588 nm. **Figure** **13**a displays fluorescence spectra of 5.0 μM of DASPC22 in pure water and in the presence of 8.1 ppb of ClO_4_^−^ ions, and in the presence of 8.0 mM of *β*‐CD with and without 8.1 ppb ClO_4_^−^ ions at pH 7.4. The threshold value is the fluorescence intensity of 940,000 that has been set in the case of the construction of logic gates 2 and 3 discussed above. Based on these results, the truth table obtained is shown in Figure 13b. On the basis of this truth table, the logic gate constructed is AND, which is shown in Figure 13c.

#### 2.3.5 Dual‐Input‐Single‐Output: Logic Gate 5

**Figure 14:**
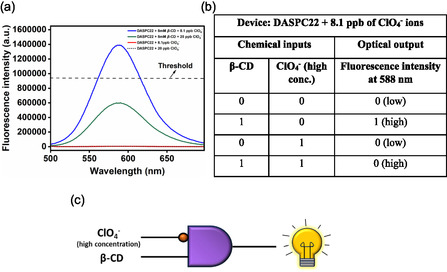
a) Fluorescence spectra of DASPC22 + 8.1 ppb ClO_4_^−^ ions, DASPC22 + 20.0 ppb ClO_4_^−^ ions, DASPC22 + 8.0 mM *β*‐CD + 8.1 ppb ClO_4_^−^ ions and DASPC22 + 8.0 mM *β*‐CD + 20 ppb ClO_4_^−^, b) truth table and c) INHIBIT logic gate representation.

The device here is the aqueous solution of a mixture of 5.0 μM concentration of DASPC22 and 8.1 ppb of ClO_4_^−^ ions at pH 7.4. Dual chemical inputs are 8.0 mM of *β*‐CD and a high concentration (20.0 ppb) of ClO_4_^−^ ions. The fluorescence intensity of DASPC22 at *λ*_em_ = 588 nm is the single optical output. The fluorescence spectra of 5.0 μM of DASPC22 in the presence of each of 8.1 and 20.0 ppb of ClO_4_^−^ ions, and in the presence of 8.0 mM of *β*‐CD and each of 8.1 and 20.0 ppb of ClO_4_^−^ ions in aqueous solutions at pH 7.4 are shown by **Figure** **14**a. With 8.1 ppb ClO_4_^−^ ions, the binding strength of DASPC22 and *β*‐CD is enhanced. However, with 20.0 ppb ClO_4_^−^ ions, as a result of the salting‐in effect, the *β*‐CD‐DASPC22 complexes are dissociated, which leads to the H‐aggregates formation of the dye. After setting a threshold value of the fluorescence intensity 940,000, the obtained truth table is displayed in Figure 14b. According to this truth table data, the INHIBIT logic gate is formed and is shown in Figure 14c.

## 3 Conclusion

The binding strength of 5.0 μM of DASPC22 with 8.0 mM of *β*‐CD in the form of nanotubes is enhanced in the presence of low concentrations of ClO_4_^−^ ions and reaches a maximum at its ≈8.1 ppb concentration. The stronger binding between DASPC22 and *β*‐CD at a low concentration range of ClO_4_^−^ ions is due to the complex formation between one ClO_4_^−^ ion and two molecules of *β*‐CD through hydrogen bonding, showing a salting‐out kind of effect. Geometry optimizations of the *β*‐CD‐DASPC22, *β*‐CD‐DASPC22‐ClO_4_^−^, and *β*‐CD‐DASPC22‐(ClO_4_^−^)_2_ complexes have been carried out using a quantum mechanics/molecular mechanics (QM/MM) model, applying a two‐level ONIOM method. The results support the stronger binding between *β*‐CD and DASPC22 in the presence of a few ClO_4_^−^ ions. The fluorescence intensity of DASPC22 starts decreasing above 8.1 ppb of ClO_4_^−^ ions as the salting‐in effect of the ions is in place. The phenomenon of enhancement of fluorescence intensity of DASPC22 in the presence of low concentration of ClO_4_^−^ ions in the range of 5.6 to at least 10.0 ppb has been used to construct a single‐input‐single‐output YES logic gate. Any unknown water sample showing a fluorescence intensity ratio higher than 1.31 in the presence of 5.0 μM of DASPC22 and 8.0 mM of *β*‐CD is safe for drinking as it has ClO_4_^−^ ions of concentration within the desirable range. A method has been proposed to get an idea of whether ClO_4_^−^ ions present in the water are outside the toxic range or not. The interfering effect due to the presence of a few other ions has been taken care of. Dual‐input‐single‐output AND and INHIBIT logic gates in the presence of low and high concentrations of ClO_4_^−^ ions as one of the inputs, respectively, have also been constructed.

## 4 Experimental Section

4.1

4.1.1

##### Materials and Methods

The dye DASPC22 and *β*‐CD were procured from Sigma Aldrich and utilized without further purification. Potassium perchlorate (KClO_4_), potassium chloride (KCl), potassium iodide (KI), sodium sulfate (Na_2_SO_4_), tri‐sodium phosphate (Na_3_PO_4_), sodium nitrate (NaNO_3_), and sodium acetate (Na_2_C_2_O_4_) were obtained from Merck, Mumbai, India, while potassium fluoride (KF) and methanol were sourced from Spectrochem. Milli‐Q water was used to prepare all aqueous solutions. The pH of the solutions was adjusted using dilute sulfuric acid and sodium hydroxide, both of which were obtained from Merck (Mumbai, India). A stock solution of DASPC22 (1.0 mM) was prepared in pure methanol for fluorescence spectroscopy studies. From which in each sample, we maintained 5.0 μM. The stock solutions of *β*‐CD, KClO_4_, KCl, KF, KI, Na_2_SO_4_, Na_3_PO_4_, NaNO_3_, and Na_2_C_2_O_4_ were prepared in Milli‐Q water, and the pH of these solutions was maintained at 7.4. To estimate the binding constant of ClO_4_^−^ ions with *β*‐CD, the samples with different concentrations of *β*‐CD and a fixed concentration of ClO_4_^−^ ions were prepared by mixing 10.0 μL of the 1.0 mM of stock solution of ClO_4_^−^ ions to the required volumes of the stock solution of *β*‐CD. Samples with varying ClO_4_^−^ concentrations and fixed DASPC22 and *β*‐CD concentrations were prepared by adding 0.01 mL of the 1.0 mM DASPC22 and 1.60 mL of 10.0 mM *β*‐CD stock solution to the required volumes of aqueous ClO_4_^−^ solutions. Similarly, solutions with varying concentrations of different salts (KClO_4_, KCl, KF, KI, Na_2_SO_4_, Na_3_PO_4_, NaNO_3_, and Na_2_C_2_O_4_) were prepared. To compare the effect of these ions in the presence and absence of ClO_4_^−^ on the fluorescence intensity of DASPC22 in the presence of *β*‐CD, samples were prepared by adding 0.01 mL of the 1.0 mM DASPC22 and 1.60 mL of 10.0 mM *β*‐CD stock solution and the required volume of each ion solution to maintain 8.1 and 10 ppb of each ion in the sample. One set of samples was prepared by mixing all the ions together, whereas the other set of samples was prepared by mixing all the ions except ClO_4_^−^. All mixtures were sonicated (LABQUEST Borosil, model USB 050) to ensure homogeneity and complete miscibility. Freshly prepared solutions were used for all fluorescence measurements. The pH of the solutions was maintained at 7.4 and monitored using an EUTECH PC 510 pH meter. All experiments were performed at room temperature (25 ± 1 °C).

##### Computational Details

Geometry optimizations of the DASPC22 and *β*‐CD complexes were carried out using a quantum mechanics/molecular mechanics (QM/MM) model, applying a two‐level ONIOM method. The DASPC22 encapsulated inside the *β*‐CD nanotube, and a ClO_4_^−^ ion on the outside of the tube were treated as the high layer, while the five *β*‐CD rings were treated as the low layer. The ωB97XD/6‐31g(d,p) level of theory was applied to the high layer, and the universal force field (UFF) was applied to the low layer. The interaction energy between the *β*‐CD nanotube and DASPC22 molecules was calculated using the supermolecule approach, and the basis set superposition error was eliminated using the counterpoise method.^[^ ^29^ ^]^ All calculations were performed using the Gaussian 16 package.^[^ ^30^ ^]^

##### UV‐Vis Absorption and Fluorescence Measurements

A careful degassing using pure N_2_ gas for 15 min was done for each stock solution before use. A JASCO UV−Vis spectrophotometer (Model: V‐650) was used for recording the absorption spectra. Fluorescence measurements were performed using a Fluorolog‐TM spectrofluorimeter (Horiba Scientific) for steady‐state emission studies. Quartz cuvettes with Teflon stoppers were utilized to minimize sample evaporation during the measurements. All fluorescence spectra were corrected for instrument sensitivity and potential inner filter effects. The slit widths for both excitation and emission monochromators were set to 3 nm. The emission spectra were recorded over the range of 433–820 nm, using an excitation wavelength of 423 nm. Further details about the instrument are found elsewhere.^[^ ^31^, ^32^ ^]^

## Conflict of Interest

The authors declare no conflict of interest.

## Author Contributions

**Anusha C. M.**: experimentation, formal analysis. **Shalini Dyagala**: methodology, experimentation, conceptualization, formal analysis, visualization, writing review and editing. **Sairathna Choppella**: quantum chemical calculations**. Mahesh Kumar Ravva**: quantum chemical calculations**. Subit Kumar Saha**: conceptualization, supervision, visualization, analysis, project administration, writing original draft & editing, resources, funding acquisition. All the authors participated in the scientific discussion.

## Supporting information

Supplementary Material

## Data Availability

The data that support the findings of this study are available from the corresponding author upon reasonable request.
