# Supplementary material for: Construction of a Fluorescence‐Based Logic Gate Seeing the Effect of Perchlorate Ions on Hemicyanine Dye–β‐Cyclodextrin Complexes to Certify Safe Drinking Water
Source: ChemistryOpen. 2025 May 4;14(10):e202500152. doi: 10.1002/open.202500152 (PMC12518029; doi:10.1002/open.202500152)
Supplement: Supplementary file 1 — Supplementary Material [file OPEN-14-e202500152-s001.pdf]

Construction of a Fluorescence-Based Logic Gate Seeing the Effect of Perchlorate Ions on Hemicyanine Dye–*β*-Cyclodextrin Complexes to Certify Safe Drinking Water

Anusha C M,[a+] Shalini Dyagala,[a+] Sairathna Choppella,[b] Mahesh Kumar Ravva,[b] and Subit Kumar Saha[a*]

[a] *Department of Chemistry, Birla Institute of Technology & Science (BITS) Pilani, Hyderabad Campus, Hyderabad, Telangana 500078, India*

[b]*Department of Chemistry, SRM University-AP, Amaravati 522240, India*

[+]Authors with equal contribution

Table S1: Comparison of reported methods for ClO_4^- ion detection with the current fluorescence-based system on DASPC22–*β*-CD complex, highlighting detection method, sensitivity, and key limitations of previous approaches.

**Earlier reports**	**Detection method**	**Detection limit (ppb)**	**Reported method**	**Advantages of the present method**
Li et *al*., 2005. Ref. 28	LC/ESI-MS/MS	0.007	Extremely sensitive but requires costly instruments (LC-MS/MS), isotope-labeled standards, expert handling.	Our system is cost-effective, field-deployable, and does not require sophisticated instrumentation.
Jackson et *al*., 1999. Ref. 29	Ion Chromatography	0.3	Requires long analysis time, expensive IC setup, possible interferences from other anions	Our fluorescence-based method is selective, real-time, and simpler to operate.
Wagner et *al*., 2007. Ref. 30	2D-Ion Chromatography	0.01	Very sensitive but costly and unsuitable for rapid or on-site testing. Requires two columns with different	Our method offers a balance of reasonable sensitivity and ease-of-use.

			affinities for the analytes.	
Xu et *al.*, 1999. Ref. 31	Electrochemiluminescence	5	Multi-step sample preparation, potential matrix effects, equipment-sensitive.	No need for extraction; operates in aqueous media with direct readout.
Alsaleh et *al.*, 2021 Ref. 32	Invertebrate biosensor	5×10^5	High detection limit; complex biological system; not suitable for routine monitoring.	Our method provides rapid, sensitive detection suitable for environmental monitoring.

References as cited in the main article:

[28] Y. Li, E. J. George, *Anal. Chem.* **2005**, *77*, 4453–4458.

[29] P. E. Jackson, M. Laikhtman, J. S. Rohrer, *J. Chromatogr. A.* **1999**, *850*, 131–135.

[30] H. P. Wagner, B. V. Pepich, C. Pohl, D. Later, K. Srinivasan, R. Lin, B. DeBorba, D. J. Munch, *J. Chromatogr. A.* **2007**, *1155*, 15–21.

[31] G. Xu, S. Dong, *Electrochem. commun.* **1999**, *1*, 463–466.

[32] S. A. Alsaleh, L. Barron, S. Sturzenbaum, *Anal. Methods* **2021**, *13*, 327–336.
